# Supplementary material for: The genome sequence of the Bicolour Sedge, Triaenodes bicolor (Curtis, 1834) (Trichoptera: Leptoceridae)
Source: Wellcome Open Res. 2026 Feb 5;11:26. Originally published 2026 Jan 12. [Version 2] doi: 10.12688/wellcomeopenres.25183.2 (PMC12993304; doi:10.12688/wellcomeopenres.25183.2)
Supplement: Peer review history

# Reviewer report: Reviewer response for version 2

**DOI:** 10.21956/wellcomeopenres.28591.r147622

**Published:** 2026-05-04

**Authors:** Panagiotis Ioannidis

**Affiliations:** 1 Foundation for Research & Technology - Hellas, Crete, Greece

The present manuscript describes the sequencing and assembly of Triaenodes bicolor, a trichopteran insect.

 The genome assembly is of high quality since virtually all generated contigs have been assigned to one of the 21  chromosome-level scaffolds. All assembly statistics are excellent; BUSCO score is at 96.1% (complete), QV is 61, and k-mer completeness is at 99.67%. Moreover, the blob plot is showing that the vast majority of the assembled contigs have a GC content around 32%, while the sequencing coverage is centered around 39x. Both of these values are the expected ones.

 The authors report that "the genome will be annotated using available RNA-Seq data...". After checking the submitted data using the provided BioProject ID, I could not find any RNAseq data associated with this particular BioProject ID. So, do the authors mean that they will use RNAseq data from other, (presumably) related species? Because that would greatly limit the sensitivity of gene prediction. It would be nice if the authors could clarify this point. And also, please don't forget to run BUSCO on the predicted gene set and compare it with the BUSCO score from the genome, in order to understand how well the gene prediction pipeline worked.

Are sufficient details of methods and materials provided to allow replication by others?

Yes

Is the rationale for creating the dataset(s) clearly described?

Yes

Are the datasets clearly presented in a useable and accessible format?

Yes

Are the protocols appropriate and is the work technically sound?

Yes

Reviewer Expertise:

bioinformatics, insect genomics

I confirm that I have read this submission and believe that I have an appropriate level of expertise to confirm that it is of an acceptable scientific standard.

# Reviewer report: Reviewer response for version 2

**DOI:** 10.21956/wellcomeopenres.28591.r148667

**Published:** 2026-03-16

**Authors:** Ricardo Rodriguez-de-la-vega

**Affiliations:** 1 Université Paris-Saclay, Gif-sur-Yvette, France

Comments on Coleman and Hrynowiecka "The genome sequence of the Bicolour Sedge..."

 In this Darwin Tree of Life (DToL) project genome note, authors describe the chromosome-level fully phased genome assemblies of a male *Triaenodes bicolor*. Raw data included HiFi and Arima-HiC v2 reads obtained with standard protocols of the Wellcome Sanger Institute (WSI) from the same specimen. The overall assembly protocol followed established methods of the WSI Tree of Life programme, including HiC-integrated hifiasm assembly, bwa-mem2 HiC mapping and YaHS scaffolding. The reported assemblies are among the highest quality among DToL releases, particulary in terms of the almost perfect assignment to chromosomal-level scaffolds for both haplotypes.

 At the time of publication, this was the 13th Trichoptera genome released by the DToL project and the first after a hiatus of over one year. The assembly presented attained a chromosome level while the other haplotype remains at the scaffold level, though the quality metrics are nearly identical. Haplotype 2 have some very large scaffolds, likely due to misjoins that may have been corrected in Haplotype 1 (manual curation is not explicitly mentioned but is common in DToL assemblies).

 One final observation:  most chromosomes in the primary assembly align to a single scaffold in the alternate haplotype assembly (using minimap2 -cx asm5), with over 90% of their length included in matching blocks. The alternate haplotype is of comparable quality to the primary assembly, though the misjoins should be addressed for downstream applications.

 I am including the links to Genomes on a Tree and Tree of Life quality check pages as I think are useful points to access the numerous underlying data analyses.

 (refer to 1,2)

Are sufficient details of methods and materials provided to allow replication by others?

Yes

Is the rationale for creating the dataset(s) clearly described?

Yes

Are the datasets clearly presented in a useable and accessible format?

Yes

Are the protocols appropriate and is the work technically sound?

Yes

Reviewer Expertise:

Genomics, Bioinformatics, Evolution

I confirm that I have read this submission and believe that I have an appropriate level of expertise to confirm that it is of an acceptable scientific standard.

## References

[1] https://goat.genomehubs.org/record?recordId.

[2] https://tolqc.cog.sanger.ac.uk/darwin/insects/Triaenodes_bicolor/.

# Reviewer report: Reviewer response for version 2

**DOI:** 10.21956/wellcomeopenres.28591.r149462

**Published:** 2026-03-06

**Authors:** Michael Hiller

**Affiliations:** 1 Senckenberg Research Institute, Frankfurt, Germany

The data note describes a high-quality chromosome-level assembly of Triaenodes bicolor. 

 All sequencing and assembly methods are described in great detail. 

 My only comment is that the contig N50 is <1 Mb, which is likely the result of GC-dependent amplification bias of the polymerases used in the original ULI protocol. I wonder if it was necessary to use the ULI protocol on a species of that size? 

 As a note, the identity of the A and B polymerases in the original ULI protocol are now known: NEBNext® High-Fidelity 2X PCR Master Mix (PacBio Ultra-Low DNA Input A) and TaKaRa Bio PrimeSTAR® GXL DNA Polymerase (PacBio Ultra-Low DNA Input B).

 This could be potentially added.

Are sufficient details of methods and materials provided to allow replication by others?

Yes

Is the rationale for creating the dataset(s) clearly described?

Yes

Are the datasets clearly presented in a useable and accessible format?

Yes

Are the protocols appropriate and is the work technically sound?

Yes

Reviewer Expertise:

comparative genomics, genome assembly

I confirm that I have read this submission and believe that I have an appropriate level of expertise to confirm that it is of an acceptable scientific standard.

# Reviewer report: Reviewer response for version 1

**DOI:** 10.21956/wellcomeopenres.27758.r144952

**Published:** 2026-01-28

**Authors:** Christophe Klopp

**Affiliations:** 1 INRAE, Castanet-Tolosan, France

The authors *provide the first assembly of Bicolour Sedge, Triaenodes bicolor. *They have used strandard procotols mixing HifI and Hi-C data and have achieved a very contiguous haplotyped assemblies.

 The busco scores presented in the snail plot seem strange because usually complete + fragmented + missing = 100%. This is not the case here. This should be checked.

 The genomescope2 and merqury kmers histograms do not present the same balance between haplotype specific and shared kmers. This is not expected. This should be checked and explained.

Are sufficient details of methods and materials provided to allow replication by others?

Yes

Is the rationale for creating the dataset(s) clearly described?

Yes

Are the datasets clearly presented in a useable and accessible format?

Yes

Are the protocols appropriate and is the work technically sound?

Yes

Reviewer Expertise:

Genome assembly and annotation

I confirm that I have read this submission and believe that I have an appropriate level of expertise to confirm that it is of an acceptable scientific standard, however I have significant reservations, as outlined above.

# Response

**Published:** 2026-01-28

**Authors:** Tree of Life Team Sanger

**Affiliations:** Wellcome Trust Sanger Institute, UK

Thank you for reviewing this article. The BUSCO values reported in the text and shown in the hosted (interactive) version of the snail plot are C: 96.1% [S: 94.8%; D: 1.3%]; F: 2.0%; M: 1.9%; *n* = 2,124, which sum to 100%.

 We identified that the static image of the snail plot originally included in the manuscript did not reflect the current BUSCO values for this dataset. This figure has now been replaced with a corrected version. GenomeScope2 analyses *read-derived* k-mer multiplicity and does not use the assembly. It models k-mer frequencies from the raw sequencing reads to infer genome properties such as heterozygosity and repeat content statistically. In contrast, MerquryFK evaluates *assembly-derived* haplotypes by partitioning read k-mers according to their presence in the assembled sequences. As these analyses use different inputs and address different questions, the relative proportions of inferred heterozygous k-mers in GenomeScope2 and haplotype-specific k-mers in MerquryFK are not expected to match. The correspondence between the heterozygous and homozygous coverage peaks inferred by GenomeScope2 and the haplotype-specific and shared peaks observed in MerquryFK nevertheless indicates consistency between the two analyses.
